# An economic experiment reveals that humans prefer pool punishment to maintain the commons

**DOI:** 10.1098/rspb.2012.0937

**Published:** 2012-07-04

**Authors:** Arne Traulsen, Torsten Röhl, Manfred Milinski

**Affiliations:** 1 Evolutionary Theory Group, Max-Planck-Institute for Evolutionary Biology, August-Thienemann-Straße 2, 24306 Plön, Germany; 2 Department of Evolutionary Ecology, Max-Planck-Institute for Evolutionary Biology, August-Thienemann-Straße 2, 24306 Plön, Germany

**Keywords:** evolution of cooperation, peer punishment, pool punishment

## Abstract

Punishment can stabilize costly cooperation and ensure the success of a common project that is threatened by free-riders. Punishment mechanisms can be classified into pool punishment, where the punishment act is carried out by a paid third party, (e.g. a police system or a sheriff), and peer punishment, where the punishment act is carried out by peers. Which punishment mechanism is preferred when both are concurrently available within a society? In an economic experiment, we show that the majority of subjects choose pool punishment, despite being costly even in the absence of defectors, when second-order free-riders, cooperators that do not punish, are also punished. Pool punishers are mutually enforcing their support for the punishment organization, stably trapping each other. Our experimental results show how organized punishment could have displaced individual punishment in human societies.

## 1. Introduction

It has been suggested that large-scale cooperation in humans is maintained because wrongdoers are punished [1,2], either by ‘peer punishment’ [3–12], where individuals decide to punish others in a dyadic way, or by ‘pool punishment’ [13–18], a kind of tax-paid organization to which punishment is outsourced. Intermediate punishment systems, where only some subjects are allowed to punish, have also been analysed [19,20]. Peer punishment is studied theoretically, experimentally and in naturally occurring environments [21] as a mechanism to stabilize cooperation in public goods games, social dilemmas in which the success of a common project is threatened by the individual temptation to free-ride on the contributions of others [22–26]. When stakes are low, we sometimes use peer punishment by personally reprimanding wrongdoers [27], a rare event in modern societies as we hardly ever observe commuters assaulting fare-dodgers or tax-payers affronting defrauders. In his *Leviathan*, Hobbes [28] suggested that the consent of people could lead to a central authority that punishes those who violate the laws of the society. At present times, these central authorities are institutions such as the police, to which punishment has been outsourced. How can such institutions emerge when they are initially inefficient [24]? In which situation is it better to rely on peer punishment and when does it pay to invest into pool punishment? When both options are available within a society, which one is preferred? We designed a behavioural experiment based on a public goods game to address these questions.

In a typical public goods game, all players can choose whether to cooperate and invest into a common pool or to defect and enjoy the benefit of the public good without investing. The invested sum is then multiplied by a constant factor and distributed among all participants. Since defecting free-riders earn more than cooperators, cooperation typically breaks down [29]. A possibility to overcome this is to give cooperators the option to peer punish and prosecute the free-riders, even if this is costly. However, there are several issues with this approach: in the short run, the efficiency cost due to peer punishment compensates or even overrides the gains from the public good [30–32], so only in the long run can peer punishment become worthwhile [8]. It works only if enough information is available [33] and the fine-to-fee ratio is high enough [34]. Counter punishment [35] or antisocial punishment [36] can lead to additional efficiency loss. Moreover, punishment itself is a second-order public good, and thus threatened by second-order free-riders who contribute to the public good, but do not punish [37–39]. Unless it can be coordinated [40], the initial emergence of a peer punishment system is problematic [41,42]. Peer punishment occurs individually, and after the public-goods interaction it resembles revenge.

Peer punishment is reactive (and may be emotional), whereas pool punishment requires planning. Pool punishers contribute ‘taxes’ to maintain a punishment system. Building up such a pool punishment system requires investments before free-riding occurs and it is costly to keep up the system even in the absence of wrongdoers [13,43]. It appears that the very nature of pool punishment is that a decision to support an organization which punishes defectors in case they show up has to be made before it is known that defectors are present. In this case, the costs to maintain the pool punishment organization (e.g. a sheriff or the police) must be independent of the later presence of defectors: it may appear to be low when many individuals are punished, but the amount is exactly the same when not a single person has to be punished. This is different from peer punishment, where each defector is punished in dyadic interactions. Thus, the cost of peer punishment is proportional to the number of people punished, but in contrast to pool punishment there is no cost when no one has to be punished. Peer punishers react to defectors directly, whereas pool punishers plan ahead and establish an organization for punishment. In its simplest form, pool punishment can be implemented by electing and paying an individual to perform the punishment [20]. Instead of allowing subjects to shape their own punishment organization, we implement the consequences of different punishment organizations that subjects can choose from. Putterman *et al.* [44] have tested voting for such formal sanction schemes for the group experimentally. They propose that one should investigate the choice between formal and informal sanctions when both are available. This approach was mathematically modelled by Sigmund *et al.* [16] and herein we test the predictions of that model experimentally.

Our basic assumptions are the same as those in the model of Sigmund *et al.* [16], which compares peer and pool punishment in a public goods game without and with second-order punishment (i.e. the punishment of those who cooperate, but do not punish). In addition to cooperators, defectors and the two forms of punishment, Sigmund *et al.* introduced loners, who abstain from the common enterprise entirely and rely on a small, but secure income [45,46]. The model compares peer and pool punishment alone incorporated into a public goods game as well as the combined availability of both forms of punishment. In summary, the model predicts that (i) the use of peer punishment is not greatly affected by the presence or absence of second-order punishment, which also has no effect on the efficiency (i.e. the average payoff of each individual in each round). (ii) Pool punishment is only used in the presence of second-order punishment, but it substantially decreases efficiency. (iii) If both punishment mechanisms are available, peer punishment is used more frequently in the absence of second-order punishment, but pool punishment prevails in the presence of second-order punishment, again with decreased efficiency. The predictions of this evolutionary model are tested experimentally herein.

## 2. Methods

We have designed experimental public goods games with volunteers to study how peer and pool punishment alone (treatments (a) and (b)) and their combination (treatment (c)) are used in the absence or presence of second-order punishment. With second-order punishment, all those who cooperate but do not punish have to pay the same fine as the defectors (cf. [3] for a discussion of this assumption). In peer punishment, second-order punishment typically implied additional costs (because additional individuals have to be punished), whereas in pool punishment this was covered by a single tax. Groups within each treatment played three consecutive public goods games; the first and second games were used to familiarize the players with each punishment regime separately, while the third game was used to provide results (table 1). Individuals could make general decisions on whether to punish a certain action, but they did not have an opportunity to target a particular individual.

**Table 1.** Overview of the experimental design. In games 1 and 2, subjects gained experience with the two punishment mechanisms in isolation, both without and with second-order punishment. Only the results of game 3 are analysed further. Treatments (a) and (b) are controls.

	rounds			treatment (a) groups		treatment (b) groups		treatment (c) groups	
	second-order punishment		initial account						
game	without	with		3 ×	3 ×	3 ×	3 ×	4 ×	4 ×
1	5	5	€ 12	peer	pool	peer	pool	peer	pool
2	5	5	€ 12	pool	peer	pool	peer	pool	peer
3	10	15	€ 24	peer	peer	pool	pool	peer and pool	peer and pool

In treatments (a) and (b), we had six groups of five subjects; in treatment (c) we had eight groups of five subjects (see table 1). The groups remained the same throughout games 1–3. Individual decisions were made in a series of yes or no questions. In each round, the players first had to choose between being a loner (fixed gain of € 0.40) and taking part in a public goods (PG) game. Those subjects deciding for the PG game can contribute either € 0 or € 0.50 to the public pool from their initial endowment of € 12 (€ 24 in game 3; see table 1). The money in the pool was multiplied by 3.1 and redistributed to all PG players. In each of the three games either peer punishment, pool punishment or combined peer and pool punishment was added to the PG game. For peer punishment, the cost for punishing was € 0.50 per punished individual, whereas the cost for being punished was € 1.00 per punisher. In pool punishment, the cost for punishing was € 0.50 and the cost for being punished was € 1.00, as in the theory paper. In all cases, punishment is costly and thus leads to an efficiency loss. As in the mathematical model, the level of efficiency depends on the cost of punishment, which can be chosen as a parameter. Therefore, the experiment could have been designed in such a way that the stable pool punishment solution is also highly efficient, but this would have precluded distinguishing whether subjects prefer the stable to the efficient solution. In peer punishment, the decision to punish (pay € 0.50 per player who did not invest to impose a fine of € 1.00) was made by the individual after they had obtained the information on contributions. In pool punishment, the decision to pay taxes for the punishment organization (pay € 0.50 such that each player who did not invest must pay € 1.00 per tax payer) had to be made before the information on contributions was available. In the experiment, we have called this organization ‘police’, because the subjects can easily relate this to real life. When both forms of punishment were combined, the pool punishment decision had to be made before the information on contributions was available, while the peer punishment decisions thereafter, however without knowledge about pool punishment decisions.

See electronic supplementary material for further details. For data requests, please contact the corresponding author.

## 3. Results

**Figure 1.**
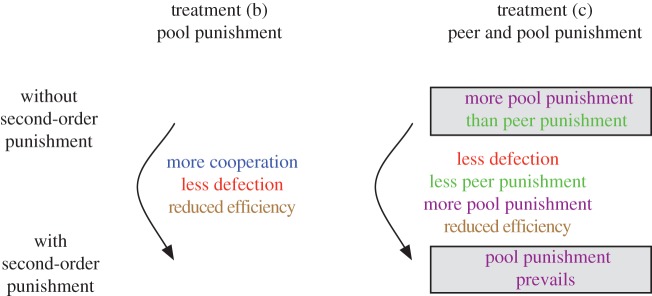
Overview over the relevant significant experimental results. In treatment (b), the introduction of second-order punishment led to a significant increase in the level of cooperation, a significant decrease in defection and a significant reduction in efficiency. In treatment (c), there is significantly more pool punishment than peer punishment in the absence of second-order punishment. If it is introduced, the level of defection and the use of peer punishment significantly decrease. The use of pool punishment significantly increases; the efficiency is reduced significantly. With second-order punishment, pool punishment prevails (i.e. the number of players using pool punishment is significantly different from 50%). Within treatment (a), the changes after the introduction of second-order punishment were not significant. See §3 for details.

In all treatments of our experiment (table 1), we found that the majority of players cooperate, both with and without second-order punishment. This is also expected from the model. An overview of all significant results is presented in figure 1.

**Figure 2.**
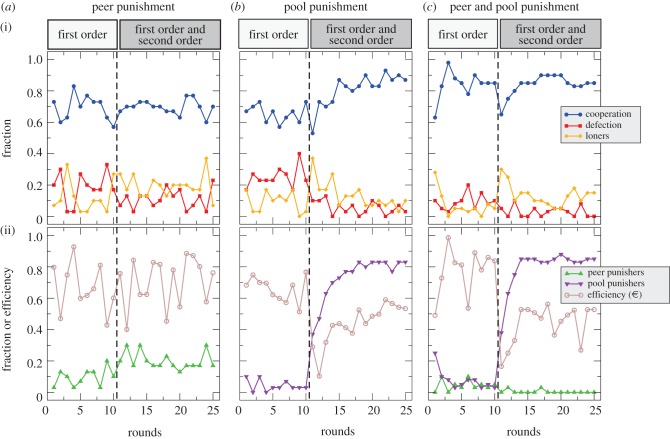
Dynamics of decision-making in game 3, which had 25 rounds. (i) Fraction of decisions to cooperate, defect or act as a loner in the three treatments (see table 1 for details). (ii) Efficiency (i.e. the average payoff in euros) per individual per round, and the fraction of players using punishment (both quantities happened to be of a similar range). The vertical lines mark the introduction of second-order punishment after round 10, which has a large impact in the experiments with pool punishment, where it reduces the efficiency significantly. In treatments (*a*) and (*c*), the average level of cooperation is slightly affected by second-order punishment only, but in treatment (*b*), second-order punishment significantly increases the level of cooperation. In isolation, pool punishment is used much more frequently than peer punishment in the presence of second-order punishment. If we combine both forms of punishment, pool punishment clearly prevails and little peer punishment is used (averages over six groups in peer punishment and pool punishment, eight groups in peer and pool punishment).

Treatment (a) considered peer punishment incorporated into the public goods game, where the subjects can postpone the punishment decision to the end of each round (see figure 2*a*). We observed no significant differences in the level of cooperation or in the efficiency between the absence and presence of second-order punishment, which corroborates the model's prediction. As in the model, the absence or presence of second-order punishment did not lead to significant differences in the frequency of defectors or loners. We observed a large fraction of cooperators that do not punish and there is little need to punish. The model's prediction is that peer punishment ‘prevails’, which means that the majority of the population adopts that strategy after some time. This can be analysed by testing whether the frequency of a strategy is above 50 per cent (i.e. whether it prevails). To answer this question, we focused on the last 10 rounds of game 3, when subjects had sufficient time to settle on a strategy. On average, the majority of the subjects used peer punishment in only 27 per cent of the rounds, which is below but not significantly different from a base value of 50 per cent (Wilcoxon one-sample test: *n* = 6 groups; we treat whole groups as statistical units and use two-tailed tests throughout, *Z* = −1.444, *p* = 0.1486). Here, the model's prediction was not supported, but if there is no defector, there is no reason to punish in the experiment see (figure 2*a*). In fact, choosing punishment in such a case does not have any effect.

Treatment (b) considered pool punishment incorporated into the public goods game (see figure 2*b*). In the absence of second-order punishment, the punishment strategy was used rarely. Without second-order punishment, there were no significant differences in the level of cooperation (Mann–Whitney *U*-test: *n*_1_ = *n*_2_ = 6, *Z* = −0.241, *p* = 0.8095) or the level of defection (Mann–Whitney *U*-test: *n*_1_ = *n*_2_ = 6, *Z* = −0.641, *p* = 0.261) between pool and peer punishment. However, once second-order punishment was added, the majority of players seemed to invest into pool punishment. This significantly increased the level of cooperation (Wilcoxon matched-pairs signed-rank test: *n* = 6, *Z* = −1.992, *p* = 0.046), and escalated the use of pool punishment (Wilcoxon matched-pairs signed-rank test: *n* = 6, *Z* = −1.992, *p* = 0.046; see figure 2*b*). The introduction of second-order punishment did not seem to influence the decisions to act as a loner, but it suppressed the number of defectors (Wilcoxon matched-pairs signed-rank test: *n* = 6, *Z* = −2.207, *p* = 0.027). However, the suppression of defection did not pay out, as second-order punishment substantially reduced the net average payoff in euros (i.e. efficiency) per individual compared with the situation without second-order punishment (Wilcoxon matched-pairs signed-rank test: *n* = 6, *Z* = −1.992, *p* = 0.046). The introduction of second-order punishment led to a loss in efficiency of about a third. Despite the increase in the use of pool punishment, it did not significantly prevail in the last 10 rounds of game 3: on average in 83 per cent of these rounds, the majority of subjects chose pool punishment, but this is not significantly different from the base value of 50 per cent (Wilcoxon one-sample test: *n* = 6, *Z* = −1.633, *p* = 0.1025).

In treatment (c), both forms of punishment were combined. As in the other treatments, the level of cooperation was high, with no significant differences between the absence and the presence of second-order punishment (see figure 2*c*). In the absence of second-order punishment the level of cooperation was significantly higher than in pool punishment alone (Mann–Whitney *U*-test: *n*_1_ = 6, *n*_2_ = 8, *Z* = −2.144, *p* = 0.032) and the level of defection was lower (Mann–Whitney *U*-test: *n*_1_ = 6, *n*_2_ = 8, *Z* = −2.591, *p* = 0.010). This effect must have resulted from the interaction between peer and pool punishment. However, the level of pool punishment was still slightly higher than the level of peer punishment (Wilcoxon matched-pairs signed-rank test: *n* = 8, *Z* = −2.047, *p* = 0.043). Once second-order punishment was introduced, the use of pool punishment increased (Wilcoxon matched-pairs signed-rank test: *n* = 8, *Z* = −2.521, *p* = 0.012; see figure 2*c*). The fraction of defectors decreased when second-order punishment was added (Wilcoxon matched-pairs signed-rank test: *n* = 8, *Z* = −2.371, *p* = 0.018). In the presence of two punishment mechanisms, the incentive to cooperate seems to be high: at the time when players decided about cooperation or defection, it was unknown if pool punishers had already committed to punish defectors. But even if no one did, there was still the option of peer punishment later. Again, second-order punishment led to a substantial loss in efficiency compared with the situation without second-order punishment (Wilcoxon matched-pairs signed-rank test: *n* = 8, *Z* = −2.521, *p* = 0.012). The level of peer punishment decreased further to almost zero (Wilcoxon matched-pairs signed-rank test: *n* = 8, *Z* = −2.366, *p* = 0.018), but this was not significantly different from the level in peer punishment alone (figure 2*a*; Mann–Whitney *U*-test: *n*_1_ = 6, *n*_2_ = 8, *Z* = −0.784, *p* = 0.4733).

In the last 10 rounds of game 3, we did not observe a single instance where at least three players used peer punishment. Pool punishment clearly prevailed: on average, in 87.5 per cent of the last 10 rounds, the majority of subjects chose pool punishment, which is significantly higher than 50 per cent (Wilcoxon one-sample test: *n* = 8, *Z* = −2.121, *p* = 0.034). When second-order punishment was added and pool punishment had been established, it was very difficult to escape contributing to pool punishment. For the group, cooperation without punishment would be a more profitable option, but it is very difficult to achieve.

## 4. Discussion

So far, the vast majority of theoretical and experimental studies on enforcement of cooperation in public goods games has been based on peer punishment. It has little effect on efficiency, because the destructive consequences of punishment occur rarely when the game is repeated for enough rounds—typically the threat of possible punishment suffices. In our case, the maximum average payoff was € 0.79 ± 0.25, which is below the theoretical optimum of € 1.05 occurring when all players cooperate but no one punishes. When only pool punishment was available, the level of cooperation was not significantly different from peer punishment without second-order punishment. Pool punishment was rarely used in this case, as expected. With second-order punishment (i.e. the punishment of cooperators who do not punish), pool punishment, however, improved the stability of cooperation, but led to a loss of efficiency (i.e. a decrease in the average payoff per individual and round) approximately € 0.51 ± 0.48. In fact, the pool punishment system was so costly that it would have been beneficial to abandon it in favour of peer punishment. In this study, every defector faced the same fine under pool punishment due to the absence of individual differences in defection.

In order to test its predictions, we had to follow the mathematical model and implemented a pool punishment mechanism that was less efficient than other more sophisticated approaches. For example, one could punish only the largest deviator in the way to give her/him a precise incentive to cooperate more, as Andreoni & Gee [17] proposed. Another approach is to assume that a small number of punishers is sufficient to achieve an optimal punishment effciency [40,47], which is similar to the volunteer's dilemma [48,49]. When both peer and pool punishment are available within the social group, both punishment options were used at a low level in the absence of second-order punishment, but their interaction significantly enhanced cooperation. In the presence of second-order punishment, pool punishers dominated and prevailed, corroborating with the model's prediction. Since thereafter any other strategy has a lower payoff, pool punishers mutually enforced each other not to deviate, and thus the situation was stable. However, ‘efficiency is traded for stability’ ([16], p. 861); stable cooperation comes at a price that reflects the fact that taxes for the organizational punishment had to be paid even in the absence of defectors. Similar to the theoretical study that motivated our experiment [16], it turned out that second-order punishment is crucial for the maintenance of pool punishment.

The major result of the corresponding theoretical model [16] that peer and pool punishment can evolve by individual selection alone was supported by our experimental findings: our players have democratically built up a pool punishment organization within their group and have forgone the opportunity to decide individually who is to be punished, as predicted. Pool punishment seemed to be a safe haven, but it came at a significant loss of efficiency. Following Hobbes, the goal of the establishment of a central authority is not to achieve the best for all, but to prevent the worst for all in a stable society.
